# Correction

**DOI:** 10.1080/14756366.2025.2537523

**Published:** 2025-07-28

**Article title:** Design, synthesis and biological evaluation of novel urolithin derivatives targeting liver cancer cells

**Authors:** Mi Tian, Lirong Zhao, Yu Lan, Chen Li, Yipeng Ling and Benhong Zhou

**Journal:** *IENZ: Journal of Enzyme Inhibition and Medicinal Chemistry*

**Bibliometrics:** Volume 40, Number 01

**DOI:** https://doi.org/10.1080/14756366.2025.2490707

When this article was first published online, there were errors in schemes 3, 4, and 5.

The correct images for the schemes are below:

Scheme 3:

**Figure:**
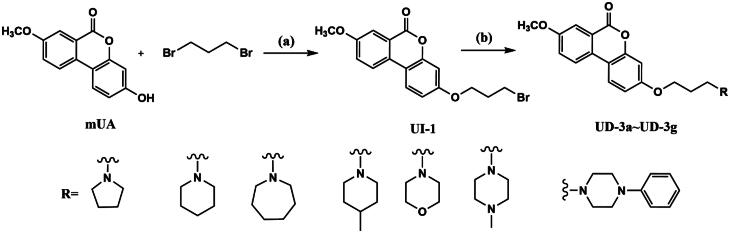

Scheme 4:

**Figure:**
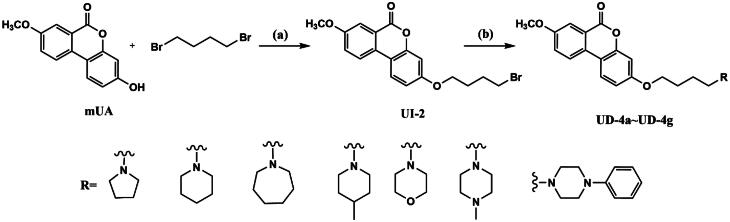

Scheme 5:

**Figure:**
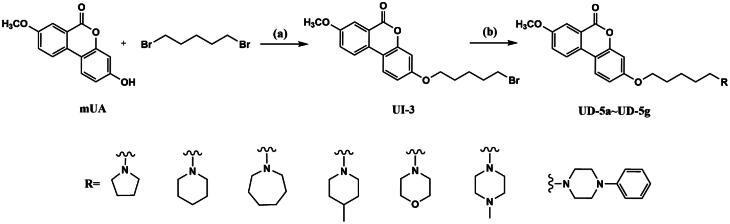

These errors have now been corrected online.

